# Supplementary material for: Participant and informant memory-specific cognitive complaints predict future decline and incident dementia: Findings from the Sydney Memory and Ageing Study
Source: PLoS One. 2020 May 12;15(5):e0232961. doi: 10.1371/journal.pone.0232961 (PMC7217434; doi:10.1371/journal.pone.0232961)
Supplement: S1 Appendix — Paraphrased SCC question followed by response options in parentheses. (DOCX) [file pone.0232961.s001.docx]

**S1 Appendix.** **Subjective complaints questions.** Paraphrased SCC question followed by response options in parentheses

|  | **Source** |
| --- | --- |
| **Participant - Memory** |  |
| Have you noticed difficulties with your memory? (yes/ no) | MAS |
| If yes to Q1, ask: Have you been concerned about your memory? (yes/ no) | MAS |
| If yes to Q1, ask: Have you mentioned any concerns about memory to anyone?  (yes/ no) | MAS |
| Have others commented on your memory not being good? (yes/ no) | MAS |
| Have you seen a GP, specialist or other health professional about your memory? (yes/ no) | MAS |
| Have you had any treatment for your memory? (yes/ no) | MAS |
| Do you now find that you lose track of what you are doing more so than 5 years ago (e.g. go into another room and can’t remember why)? (no or less/ same/ little more/ lot more) | MAS |
| Compared to 5 years ago, do you now have more difficulty remembering appointments? (no or less/ same/ little more/ lot more) | MAS |
| Compared to 5 years ago, do you now have more difficulty remembering names? (no or less/ same/ little more/ lot more) | MAS |
| Compared to 5 years ago: Remembering the name of a person just introduced to you (much better/ somewhat better/ about same/ somewhat worse/ much worse) | MAC-Q |
| Compared to 5 years ago: Recalling telephone numbers or post codes that you use on a daily or weekly basis? (much better/ somewhat better/ about same/ somewhat worse/ much worse) | MAC-Q |
| Compared to 5 years ago: Recalling where you have put objects (such as keys) in your home or office? (much better/ somewhat better/ about same/ somewhat worse/ much worse) | MAC-Q |
| Compared to 5 years ago: Remembering specific facts from a newspaper or magazine article that you have just finished reading? (much better/ somewhat better/ about same/ somewhat worse/ much worse) | MAC-Q |
| Compared to 5 years ago: Remembering the item/s you intended to buy when you arrive at the supermarket store or pharmacy? (much better/ somewhat better/ about same/ somewhat worse/ much worse) | MAC-Q |
| In general, how would you describe your memory as compared to 10 years ago? (much better/ somewhat better/ about same/ somewhat worse/ much worse) | MAC-Q |
|  |  |
| **Participant – Non-memory** |  |
| Compared to 5 years ago, do you have more difficulty finding the right words (e.g. feeling like the word is on the tip of your tongue?) (no or less/same/little more/lot more) | MAS |
| Compared to 5 years ago, do you now have more difficulty following a conversation (not due to hearing loss)? (no or less/same/little more/lot more) | MAS |
| Compared to 5 years ago, do you now have more difficulty explaining things? (no or less/same/little more/lot more) | MAS |
| Compared to 5 years ago, do you have more difficulty organizing or planning something (e.g. following a recipe, paying bills on time)? (no or less/same/little more/lot more) | MAS |
| Compared to 5 years ago, do you have more difficulty solving problems? (e.g. when a household appliance breaks down, or the kitchen sink is blocked)? (no or less/same/little more/lot more) | MAS |
| Compared to 5 years ago, do you now have more difficulty or less interest in your usual activities (e.g. hobbies, social outings)? (no or less/same/little more/lot more) | MAS |
| Compared to 5 years ago, do you now have more difficulty finding your way around familiar places (e.g. your local neighbourhood)? (no or less/same/little more/lot more) | MAS |
| Compared to 5 years ago, do you now have more difficulty finding your way around unfamiliar places (e.g. somewhere you go infrequently outside of your neighbourhood)? (no or less/same/little more/lot more) | MAS |
| Compared to 5 years ago, do you now have more trouble getting dressed (e.g. getting the buttons wrong, or putting something on inside out)? (no or less/same/little more/lot more) | MAS |
| **Informant – Memory** |  |
| Have you noticed the participant having difficulty with his/her memory? (yes/no) | MAS |
| If yes to Q1, ask: Have you been concerned about participant’s memory? (yes/no) | MAS |
| If yes to Q1, ask: Have you mentioned concerns about participant’s memory to anyone (including him/her)? (yes/no) | MAS |
| Does *[the participant]* have more trouble remembering things that have happened recently? (yes/no) | GP-Cog |
| Does *[the participant]* have more trouble recalling conversations a few days later? (yes/no) | GP-Cog |
| Compared to 5 years ago, how is the participant at remembering things about family and friends (e.g. occupations, birthdays, addresses)? (much improved/ a bit improved/ not much change/ a bit worse/ much worse) | IQCODE |
| Compared to 5 years ago, how is the participant at remembering things that happened recently? (much improved/ a bit improved/ not much change/ a bit worse/ much worse) | IQCODE |
| Compared to 5 years ago, how is the participant at recalling conversations a few days later? (much improved/ a bit improved/ not much change/ a bit worse/ much worse) | IQCODE |
| Compared to 5 years ago, how is the participant at remembering his/her address and telephone number? (much improved/ a bit improved/ not much change/ a bit worse/ much worse) | IQCODE |
| Compared to 5 years ago, how is the participant at remembering what day and month it is? (much improved/ a bit improved/ not much change/ a bit worse/ much worse) | IQCODE |
| Compared to 5 years ago, how is the participant at remembering where things are usually kept? (much improved/ a bit improved/ not much change/ a bit worse/ much worse) | IQCODE |
| Compared to 5 years ago, how is the participant at remembering where to find things which have been put in a different place from usual? (much improved/ a bit improved/ not much change/ a bit worse/ much worse) | IQCODE |
| Compared to 5 years ago, how is the participant at knowing how to work familiar machines around the house? (much improved/ a bit improved/ not much change/ a bit worse/ much worse) | IQCODE |
| Compared to 5 years ago, how is the participant at learning how to use a new gadget or machine around the house? (much improved/ a bit improved/ not much change/ a bit worse/ much worse) | IQCODE |
| Compared to 5 years ago, how is the participant at learning new things in general? (much improved/ a bit improved/ not much change/ a bit worse/ much worse) | IQCODE |
|  |  |
| **Informant – Non-memory** |  |
| When speaking, does *[the participant]* have more difficulty in finding the right word or tend to use the wrong words more often? (yes/ no) | GP-Cog |
| Compared to 5 years ago, how is the participant at following a story in a book or on TV? (much improved/ a bit improved/ not much change/ a bit worse/ much worse) | IQCODE |
| Compared to 5 years ago, how is the participant at making decisions on everyday matters? (much improved/ a bit improved/ not much change/ a bit worse/ much worse) | IQCODE |
| Compared to 5 years ago, how is the participant at using his/her intelligence to understand what's going on and to reason things through? (much improved/ a bit improved/ not much change/ a bit worse/ much worse) | IQCODE |

*Paraphrased SCC question followed by response options in parentheses
