# Supplementary material for: Participant and informant memory-specific cognitive complaints predict future decline and incident dementia: Findings from the Sydney Memory and Ageing Study
Source: PLoS One. 2020 May 12;15(5):e0232961. doi: 10.1371/journal.pone.0232961 (PMC7217434; doi:10.1371/journal.pone.0232961)
Supplement: S2 Appendix — Diagnostic criteria used for normal, MCI and dementia consensus diagnoses. (DOCX) [file pone.0232961.s002.docx]

**S2 Appendix. Explanatory Notes for MAS Diagnostic Classifications.** Diagnostic criteria used for normal, MCI and dementia consensus diagnoses.

**MCI Classification**

Participants were classified as having MCI according to international consensus criteria ([Petersen 2004](#_ENREF_12)) and if all of the following criteria were met:

1. A subjective complaint of decline in memory or other cognitive function (from the participant and/or their informant)
2. Cognitive impairment as shown by performance 1.5 standard deviations (or equivalent) below published normative values (matched for age and education where available) on a neuropsychological test measure
3. Normal or minimally impaired functional activities as determined by informant ratings on the Bayer-ADL scale ([Hindmarch, Lehfeld et al. 1998](#_ENREF_5)). This is a questionnaire completed by the participant’s informant that rates their level of difficulty in instrumental activities of daily living.
4. Not demented i.e.no diagnosis of DSM-IV dementia ([APA 2000](#_ENREF_1)), as determined by a consensus diagnosis from an expert team comprised of old age psychiatrists, neuropsychiatrists and neuropsychologists.

**Normal Classification**

Normal performance on neuropsychological test measures; above -1.5 SDs compared to published normative values (matched for age and education where available).

**Dementia Classification**

The diagnosis of dementia was made by an expert panel of clinicians including old age psychiatrists, neuropsychiatrists and neuropsychologists using information from a clinical interview, comprehensive neuropsychological test battery, informant-based report of instrumental activities of daily living and neuropsychiatric symptoms and MRI when available (approximately half the sample). The diagnosis of dementia was based on DSM-IV criteria ([APA 2000](#_ENREF_1))- that is, the presence of multiple cognitive deficits that represent a decline from a previous level of functioning and include memory impairment and at least one other cognitive disturbance. The cognitive deficits are sufficiently severe to cause impairment in functioning. Dementia subtype is listed for cases for which this could be determined.
