# Supplementary material for: Participant and informant memory-specific cognitive complaints predict future decline and incident dementia: Findings from the Sydney Memory and Ageing Study
Source: PLoS One. 2020 May 12;15(5):e0232961. doi: 10.1371/journal.pone.0232961 (PMC7217434; doi:10.1371/journal.pone.0232961)
Supplement: Attachment — Submitted filename: Response to Reviewers.docx [file pone.0232961.s005.docx]

April 7^th^, 2020

Dear Academic Editor,

Thank you for your positive appraisal of our manuscript titled “Participant and informant memory-specific complaints predict future decline and incident dementia: Findings from the Sydney Memory and Ageing Study”.

Reviewer #1: The study presents the results of original research. The findings of the manuscript are already well-known, but data presented here help put what is known into a little more focus (patients perceive language difficulties, family perceives executive problems). Experiments, statistics, and other analyses are performed to a high technical standard and are described in sufficient detail. Conclusions are presented in an appropriate fashion. The article is presented in an intelligible fashion and is written in standard English. The research meets all applicable standards for the ethics of experimentation and research integrity. The article adheres to appropriate reporting guidelines and community standards for data availability.

Just one thing worth addressing that does not quite make sense and which is important to the logic of this study: if informants are corroborating the presence of deficits, then aren't the deficits by definition objective rather than subjective?

Reviewer #2: I believe this is the first study to examine the relationship between participant-reported and informant-reported memory and non-memory SCCs, cognitive decline and incident dementia over six years. The methods are well executed and well written manuscript.

- Regarding use of the term “subjective” when others have also reported cognitive decline, we point out that we use this term in line with MCI consensus criteria (Winblad et al., 2004), DSM-5 definitions (American Psychological Association, 2013) and the more recently published SCD consensus criteria paper (Jessen et al., 2014)*.* “Objective impairment”, by convention, is determined by performance on validated psychometric tools using established cut-off scores adjusted for age and education, where “subjective impairment” is any self-reported or other-reported impression of impairment or decline.
- The comment by Reviewer 1 also suggests that the participants’ complaints were corroborated by the responses of their informants, however, we examined informant and participant complaints and their respective predictive values separately in this paper. That is, no analyses were carried out to determine whether there was a high level of agreement between informants’ and participants’ subjective evaluations of cognitive ability. Although we agree that examining consensus between informant and participant evaluations is an interesting research question, and worthy of investigation in a future paper based on this cohort, in the present paper no analyses were carried out to examine whether this was, in fact, the case. For these reasons, we have not amended the manuscript.

Please ensure that your manuscript meets PLOS ONE's style requirements, including those for file naming.

- We have updated the format of our manuscript to ensure it meets all PLOS ONE’s style requirements. Please see attached revised manuscript with track changes.

If there are ethical or legal restrictions on sharing a de-identified data set, please explain them in detail (e.g., data contain potentially sensitive information, data are owned by a third-party organization, etc.) and who has imposed them (e.g., an ethics committee). Please also provide contact information for a data access committee, ethics committee, or other institutional body to which data requests may be sent.

- Although we are happy to provide aggregated/summary-level data, the ethics approval for our research (provided by UNSW HREC, ref number HC190962) does not allow for unmediated access to participant-level data. The terms of consent for research participation stipulate that an individual's data can only be shared outside of the MAS investigators group if the group has reviewed and approves the proposed secondary use of the data. This consent applies regardless of whether data has been de-identified. Access is mediated via a standardised request process managed by the CHeBA Research Bank, who can be contacted at [ChebaData@unsw.edu.au](mailto:ChebaData@unsw.edu.au), or via the first author's contact details at [k.numbers@unsw.edu.au](mailto:k.numbers@unsw.edu.au).

If there are no restrictions, please upload the minimal anonymized data set necessary to replicate your study findings as either Supporting Information files or to a stable, public repository and provide us with the relevant URLs, DOIs, or accession numbers

- Please see response to prompt above.

Please amend either the title on the online submission form (via Edit Submission) or the title in the manuscript so that they are identical.

- We have edited the title of the manuscript so that it is identical to that of the online submission.

Please amend either the abstract on the online submission form (via Edit Submission) or the abstract in the manuscript so that they are identical.

- We have edited the abstract in the manuscript so that it is identical to the online submission.

[Please include captions for your Supporting Information files at the end of your manuscript, and update any in-text citations to match accordingly.](http://journals.plos.org/plosone/s/supporting-information)

- We have included captions for all Supporting Information files, which now appear after References, and have updated intext citations accordingly.

Again, we thank you for your time and consideration and look forward to receiving your final decision.

Sincerely,

Katya Numbers, PhD

E: [k.numbers@unsw.edu.au](mailto:k.numbers@unsw.edu.au)

M: +61 0459 613 288
